# Zinc suppresses Stat3-driven IL-6 production in primary mouse adipocytes

**DOI:** 10.3389/fimmu.2026.1714168

**Published:** 2026-02-09

**Authors:** Hak Chung, John Eom, Michelle SMA Damen, Traci E. Stankiewicz, Keisuke Sawada, Pablo C. Alarcon, Cassidy J. Ulanowicz, Jennifer L. Wayland, George S. Deepe, Senad Divanovic

**Affiliations:** 1 Division of Immunobiology, Cincinnati Children’s Hospital Medical Center, Cincinnati, OH, United States; 2 Department of Pediatrics, University of Cincinnati College of Medicine, Cincinnati, OH, United States; 3 Immunology Graduate Program, University of Cincinnati College of Medicine, Cincinnati, OH, United States; 4 Medical Scientist Training Program, University of Cincinnati College of Medicine, Cincinnati, OH, United States; 5 Division of Infectious Diseases, University of Cincinnati College of Medicine, Cincinnati, OH, United States; 6 Center for Inflammation and Tolerance, Cincinnati Children’s Hospital Medical Center, Cincinnati, OH, United States

**Keywords:** adipose tissue, cytokines, immunity, metals, obesity, pyrithione

## Abstract

Uncontrolled inflammatory cytokine production promotes pathogenesis of various chronic diseases. Zinc (Zn) regulates immune cell inflammatory cytokine production. However, the influence of Zn on the inflammatory properties of non-immune cells known to contribute to disease pathogenesis is not well understood. Adipocytes respond to various immunological stimuli by activating inflammatory pathways and secreting inflammatory cytokines. Here, we investigated the impact of Zn on adipocyte inflammatory vigor. We show that treatment of primary mouse adipocytes with Zn, in the form of Zn pyrithione, restricted their toll-like receptor ligand-driven IL-6 production. Mechanistically, IL-6 secreted from adipocytes functions in an autocrine fashion to activate the Stat3 pathway and amplify IL-6 production via a positive feedback loop. Notably, Zn treatment of adipocytes suppressed Stat3 signaling activation to break the positive feedback loop and subsequent expression of IL-6 and its receptor genes (*Il6st*, *Il6ra*). Collectively, our findings uncover a novel inhibitory role for Zn in non-immune cell, specifically adipocyte, IL-6 production. These findings invoke a potential role of Zn in the regulation of adipocyte-associated chronic inflammation and disease pathogenesis.

## 1 Introduction

Inflammation is a multifaceted immunological process that is integral to the body’s defense mechanisms against injury and external threats. This natural and essential biological response is vital for providing protection from environmental challenges. Nevertheless, non-resolving or chronic inflammation can lead to numerous health complications, often serving as a common underlying factor in the pathogenesis of various diseases (1). Therefore, tight regulation of inflammation is required to mitigate unwanted disease states.

Chronic inflammation is often marked by an uncontrolled release of inflammatory cytokines, including Interleukin-6 (IL-6) (2). IL-6, a pleotropic cytokine produced by both immune and non-immune cell types, affects immune system homeostasis, hematopoiesis, inflammation, and metabolism (3, 4). Cellular sensing of IL-6 occurs through activation of the gp130 signal transducer, initiated by the binding of IL-6 to the IL-6 receptor (IL-6R) present in either transmembrane or soluble forms (5). Chronic elevation in IL-6 is observed in multiple pathological conditions. In obesity, for example, systemic and local IL-6 is strongly associated with the development of metabolic disorders. In fact, IL-6 is recognized as an initiator of hepatocyte insulin resistance (6) and promoter of atherosclerosis (7). Further, IL-6 is essential for the differentiation of CD4^+^ T cells into Th17 cells (8) and sustaining pathogenic Th17 cell subset (9), both of which are considered primary culprits of obesity-associated liver inflammation (10, 11).

Among various non-immune cells, adipocytes are appreciated to produce large amounts of IL-6 (12). Adipocytes are traditionally viewed as energy storing cells due to their remarkable lipid storage capacity; however, their immune-like properties have recently been recognized (12–14). Adipocytes express various immune receptors (e.g., toll-like receptors (TLRs), type I interferon receptor, etc.) and are capable of producing significant amounts of inflammatory cytokines including IL-6 (15). In humans, up to 35% of basal circulating IL-6 originates from white adipose tissue (WAT), with both immune cells and adipocytes in WAT serving as major sources of IL-6 (16). Although debatable, a study reported that adipocyte-specific IL-6 knockout mice fed a high-fat diet had ~40% decrease in circulating IL-6 levels (17), invoking the potential impact of adipocyte-centric IL-6 production to disease pathogenesis.

Research efforts to elucidate the exact role of IL-6 in the development of metabolic disorders involving chronic inflammation, however, have revealed conflicting findings. In fact, a substantial body of evidence suggests beneficial metabolic roles of IL-6 (18) – whole-body IL-6 knockout mice develop obesity, liver inflammation and insulin resistance on standard chow diets (19), and skeletal muscle-derived IL-6 during exercise acts as an insulin sensitizer (20). IL-6 is also recognized for its ability to drive thermogenic programming and browning during adipocyte development (21). In addition, an anti-inflammatory role of IL-6 promoting M2-like polarization of adipose tissue macrophages (ATMs) has been reported (22). These contradictory findings demonstrate the complexity of IL-6 functions in chronic inflammation and suggest that IL-6 role is likely highly context-dependent. Importantly, Han et al. (17) revealed that the cellular origin of IL-6 is critical in determining its effects: myeloid cell-derived IL-6 suppresses M1-like macrophage polarization, limits ATM accumulation, and improves glucose and insulin tolerance, whereas adipocyte-derived IL-6 strongly promotes ATM accumulation and exacerbates high-fat diet-induced adipose tissue inflammation. These results highlight the potential of limiting adipocyte-specific IL-6 release for controlling chronic inflammation, and warrant further research into the regulation of adipocyte production of IL-6.

The immune system and its function are influenced by numerous factors, one of which is zinc (Zn). Zn is the second most prevalent transient metal in the human body. The Zn proteome comprises an estimated 3,000 proteins found in all six classes of enzymes, underscoring its significance in the overall cellular metabolism (23). Zn is indispensable for the proper functioning of cells, serving structural, catalytic, and signaling roles (24, 25). In the immune system, disrupted Zn homeostasis compromises both innate and adaptive immune responses and alters key immune functions such as chemotaxis, cell adhesion, phagocytosis, cytotoxicity, differentiation, proliferation, maturation, and cytokine and antibody production [reviewed in (26)]. Indeed, dysregulated Zn status is often observed in chronic inflammatory settings including arthritis, asthma, inflammatory bowel disease, and systemic lupus erythematosus (27). In obesity, Zn deficiency is prevalent as well (28) and low Zn intake or serum levels correlate with increased levels of inflammatory cytokines (29, 30). Zn supplementation trials in this population report favorable effects such as weight loss (31) and improved insulin sensitivity (32, 33), along with decreased levels of serum high-sensitivity C-reactive protein (hs-CRP) and IL-6 (34, 35). In addition, Zn supplementation decreased serum alanine aminotransferase (ALT) and CRP levels in children with non-alcoholic steatohepatitis (NASH; recently renamed as metabolic dysfunction-associated steatohepatitis, or MASH) (36), suggesting the potential of replenishing Zn in mitigating inflammation-driven pathologies (37).

Given the pivotal role of Zn in immune regulation, the immune-like properties of adipocytes, and disrupted Zn homeostasis in chronic inflammatory state, we hypothesized that Zn plays an important regulatory role in adipocyte inflammatory cytokine production. Thus, we focused our investigation on whether and how Zn modulates adipocyte IL-6 production. In this study, using primary wildtype (WT) mouse adipocytes isolated and differentiated from the stromal vascular fraction (SVF) of inguinal WAT, we demonstrate that Zn pyrithione (ZnPT) treatment restricts TLR-driven IL-6 production in adipocytes. Mechanistically, ZnPT treatment was sufficient to suppress the expression of IL-6 receptors and inhibited Stat3 signaling without altering adipocyte viability. Further, we show that IL-6 produced by adipocytes operates in an autocrine fashion to activate the Stat3 pathway and amplify IL-6 production via positive feedback loop. Together, our results demonstrate a novel inhibitory role of Zn in adipocyte IL-6 production and suggest a potential role for Zn in the regulation of adipocyte-associated chronic inflammation.

## 2 Materials and methods

### 2.1 Mice and primary adipocytes

All mice used were WT C57BL/6J, originally purchased from Jackson Laboratories, housed and maintained in a specific pathogen-free facility at Cincinnati Children’s Hospital Medical Center in accordance with Guide for the Care and Use of Laboratory Animals. All studies were approved by the Cincinnati Children’s Hospital Medical Center Institutional Animal Care and Use Committee (IACUC; protocol number 2023-0015). Free access to autoclaved chow diet (LabDiet #5010) and water was provided with weekly replacement.

Primary mouse adipocytes were generated by differentiating the stromal vascular fraction (SVF) isolated from inguinal white adipose tissue (iWAT) as previously described (12, 38, 39). iWAT was minced and digested with collagenase type IV (Gibco #17104019; 1 mg/mL) and dispase II (Roche #04942078001; 3 U/mL) in PBS with 10 mM CaCl_2_. The SVF containing preadipocytes was isolated and cultured until confluence in preadipocyte growth media (GM; 10% FBS, 100 U/mL Penicillin, 100 mg/L Streptomycin, 2 mM L-Gln in DMEM/F-12) with fresh media provided every two days. When confluence was reached at 5~6 days post-seeding, differentiation was induced by subjecting the cells to induction media (0.5 mM 3-Isobutyl-1-methylxanthine, 1 μM dexamethasone, 1 μM rosiglitazone and 850 nM insulin in GM), followed by continuation media (rosiglitazone and insulin in GM), and differentiation media (insulin in GM), each for two days. At day 6 post-induction, maximal differentiation was confirmed under microscope prior to performing experiments.

### 2.2 Treatment of cells and cytokine quantification

Cells were exposed to zinc pyrithione (ZnPT; 2 μM unless otherwise indicated), zinc chloride (ZnCl_2_), zinc sulfate (ZnSO_4_), sodium pyrithione (NaPT; 4 μM), and/or *N,N,N’,N’*-tetrakis-(2-pyridylmethyl)ethylenediamine (TPEN; 5 μM) diluted into fresh GM at indicated concentrations for three hours. Cells were then stimulated with LPS (100 ng/mL), Pam2CSK4 (10 μg/mL), poly(I:C) (25 μg/mL), flagellin (1 μg/mL), PMA (50 ng/mL) + ionomycin (1 μg/mL), PHA (10 μg/mL), αCD3 (1 μg/mL), or recombinant mouse IL-6 (rIL-6; Life Technologies #14806162; 1 μL/mL) for four hours or for specified time after stimulation. Cells treated with zinc compounds and immune stimulants were paralleled by control groups that received mock treatment with vehicles. Time point of 0 min indicates sample collection before stimulation, whereas 0+ min indicates immediately after. For Stat3 inhibition experiments, cells were exposed to ZnPT or vehicle control (DMSO) for three hours, followed by Stat3 inhibitor C188-9 (Selleckchem #S8605; 20 μM) treatment together with LPS stimulation. Culture media was collected for quantification of IL-6 cytokine levels by ELISA as per manufacturer’s instruction (BD OptEIA).

### 2.3 Quantitative RT-PCR

Primary adipocytes, cultured as described above, were homogenized in TRIzol (Invitrogen) and RNA was extracted, followed by reverse transcription using Verso cDNA Synthesis Kit (Thermo Scientific). Quantitative real-time PCR was conducted using PerfeCTa qPCR SuperMix (VWR) on QuantStudio 7 (Applied Biosystems). All procedures followed the manufacturers’ instructions. Data was analyzed using ΔΔCt method, and the mRNA expression of indicated genes were quantitated using *Actb* as normalization control. The sequence of primer pairs used is listed in **Supplementary Table 1**.

### 2.4 RNA-seq analysis

Primary adipocytes isolated and differentiated above, and bone marrow-derived macrophages isolated from femur as previously described (40–43), were stimulated with or without LPS (100 ng/mL) for four hours, and gene expression profile was determined as previously described (12). Briefly, RNA was extracted, quantified (Qubit RNA Assay Kit), and library was prepared (Illumina TruSeq RNA Library Preparation Kit), which was subjected to sequencing by 50 base pair single-end reads (~20 million reads per sample). Transcriptomic analysis was performed using AltAnalyze software (ver 2.1.4.3) (44). Reasonably expressed transcripts were subjected to differential expression analysis (unstimulated versus stimulated) through *t*-test with *p*-values adjusted for multiple testing using the Benjamini-Hochberg (BH) false discovery rate approach. *p*-value cutoff of 0.05 and fold change requirement of >1.5 were applied. Pathway enrichment analysis was performed using ToppGene with KEGG pathways for mouse.

### 2.5 Protein analysis by Western blotting

Cells were lysed in RIPA buffer with protease inhibitor (Roche cOmplete) and phosphatase inhibitor (Pierce) cocktails, and cell debris was removed after centrifugation. Total protein was quantified by Bradford assay (Bio-Rad) to equalize protein concentration across treatment conditions. Samples were mixed with Laemmli buffer (Bio-Rad) containing fresh β-mercaptoethanol (2.5% final), boiled for 5 mins, and loaded onto Mini-PROTEAN TGX Precast Gels (4-15%) at 10-20 μg/well, to be separated by SDS-PAGE in running buffer (25 mM Tris, 192 mM glycine, 0.1% SDS). Proteins were then transferred to 0.45 μm pore sized PVDF membrane in CAPS transfer buffer (10 mM CAPS, 10% ethanol, pH 11). Membranes were blocked with 5% skim milk/TBST (0.1% Tween 20) for 1 hour and incubated with respective primary antibody overnight at 4°C, followed by 2-hour incubation with secondary antibody (anti-rabbit IgG HRP, Cell Signaling Technology #7074; 1:5000 in 5% milk/TBST), all with gentle agitation. Primary antibodies used are Erk1/2 (137F5) Rabbit mAb (Cell Signaling Technology #4695; 1:500 in 5% milk/TBST), phospho-Erk1/2 (Thr202/Tyr204) (D13.14.4E) XP Rabbit mAb (Cell Signaling Technology #4370; 1:500 in 2.5% milk + 2.5% BSA/TBST), Stat3 (C-20) (Santa Cruz #sc-482; 1:1000 in 5% milk/TBST), p-Stat3 (Ser727)-R (Santa Cruz #sc-8001-R; 1:1000 in 5% milk/TBST), and α-Tubulin (11H10) Rabbit mAb #2125; 1:3000 in 5% milk/TBST). Protein signals were detected by chemiluminescence (Thermo Scientific #34580) following manufacturer’s instruction with Bio-Rad ChemiDOC Touch imaging system. Densitometry analysis was performed with ImageJ software.

### 2.6 Live cell imaging

For visualization of intracellular free Zn ion, primary adipocytes were incubated with 1 µM FluoZin-3 AM (Invitrogen) for 1 hour at 37°C. Cells were washed twice with PBS and incubated in fresh, dye-free media for additional 30 min. Images were acquired 5 min post-treatment with 2 µM ZnPT using Keyence BZ-X810 microscope with FITC filter. For cell viability assessment, adipocytes were pre-treated with or without 2 µM ZnPT for 3 hours, followed by stimulation with LPS for additional 4 hours. Cells were stained with 0.4% Trypan Blue solution and brightfield images were captured. For all images, 100 µm scale bar was applied for reference on bottom right corner.

### 2.7 Statistical analysis

All experiments were performed with two or more independent biological repeats. Data are presented as means ± SEM, with individual data points included when applicable. Data analysis was performed using GraphPad Prism software. Statistical significance was tested by Student’s *t*-test (unpaired, non-parametric, two-tailed) for comparisons between two groups, or one-way ANOVA followed by Bonferroni *post hoc* analysis for comparisons between three or more groups (α=0.05). The level of significance is denoted using asterisks, with corresponding *p*-value thresholds indicated in figure legends.

## 3 Results

### 3.1 Adipocytes have immune cell-like properties

**Figure 1:**
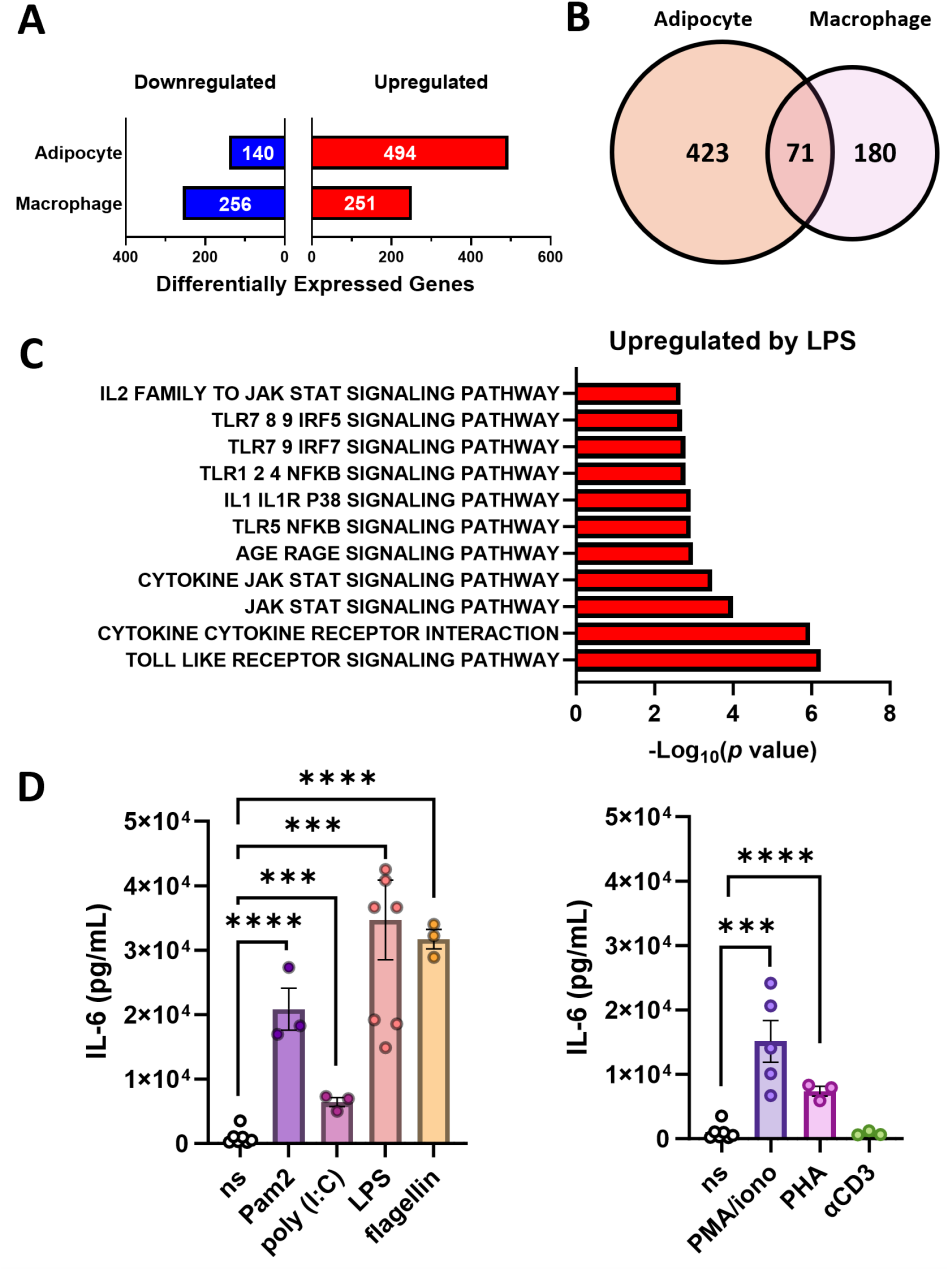
TLR-driven induction of inflammatory signaling pathways in adipocytes. **(A–C)** Gene expression analysis performed using AltAnalyze (n=2 biological replicates per group). **(A)** Genes that have greater than 1.5-fold difference in expression between unstimulated and LPS-stimulated adipocytes or macrophages (red, upregulated; blue, downregulated) with *p* < 0.05 significance by unpaired *t*-test (multiple testing corrected via BH method). **(B)** Number of genes that are upregulated in either adipocytes, macrophages, or both. **(C)** ToppGene pathway enrichment analysis of the 71 co-upregulated genes in LPS-stimulated adipocytes and macrophages using KEGG pathways for mouse. From the 20 most significantly altered pathways, those related to inflammatory response and cytokine production are selectively displayed. Full list of differentially expressed genes is provided in **Supplementary Datasheet 2**. **(D)** IL-6 levels in the primary adipocyte culture media upon TLR-specific stimulation (left panel) or general immunological stimulation (right panel) measured by cytokine ELISA. ns, non-stimulated. Bars and error bars represent means ± SEM, circles represent independent repeats. Asterisk indicates statistical significance by Student’s *t*-test (***p<0.001, ****p<0.0001).

To begin to broadly characterize adipocyte inflammatory potential, we stimulated primary mouse adipocytes or bone marrow-derived macrophages with vehicle or LPS, and compared their gene expression profiles using bulk RNA-Seq analysis. Differentially expressed genes in LPS-stimulated adipocytes and macrophages, relative to unstimulated counterparts, were identified. Notably, LPS stimulation substantially altered gene expression in both adipocytes and macrophages (**Figure 1A**). Among 494 genes that were upregulated by LPS stimulation in adipocytes, 71 were also increased by LPS stimulation in macrophages (**Figure 1B**). This finding indicates a conserved LPS-induced transcriptomic responses between adipocytes and macrophages. Pathway enrichment analysis revealed that the 71 genes co-upregulated in LPS-stimulated adipocytes and macrophages are largely relevant to cellular inflammatory responses and cytokine production (**Figure 1C**). Further, LPS stimulation resulted in downregulation of a set of genes in adipocytes, although their conservation with genes downregulated in LPS-stimulated macrophages was highly limited (**Supplementary Figure 1**). These data confirm that adipocytes, akin to macrophages, have the capacity to sense inflammatory stimuli and respond by adjusting cellular processes via alterations in their transcriptional profile.

To examine whether adipocyte inflammatory capability is restricted to LPS, primary mouse adipocytes were stimulated with various immune activators and their ability to produce inflammatory cytokines was evaluated. Of note, as adipocytes are robust producers of IL-6 (16), we focused our attention on adipocytes’ ability to produce IL-6. Stimulation with various TLR-specific ligands (e.g., Pam2CSK4, poly(I:C), and flagellin) or common immune activators (e.g., phorbol myristate acetate [PMA]/ionomycin, phytohemagglutinin [PHA]), were all sufficient to induce IL-6 production in adipocytes (**Figure 1D**). Exposure to anti-CD3, a selective activator of T cell receptor signaling, did not elicit IL-6 production by adipocytes. Together, these results highlight wide-ranging immune cell-like properties of adipocyte inflammatory responses.

### 3.2 Zinc restricts TLR-driven IL-6 production of adipocytes

**Figure 2:**
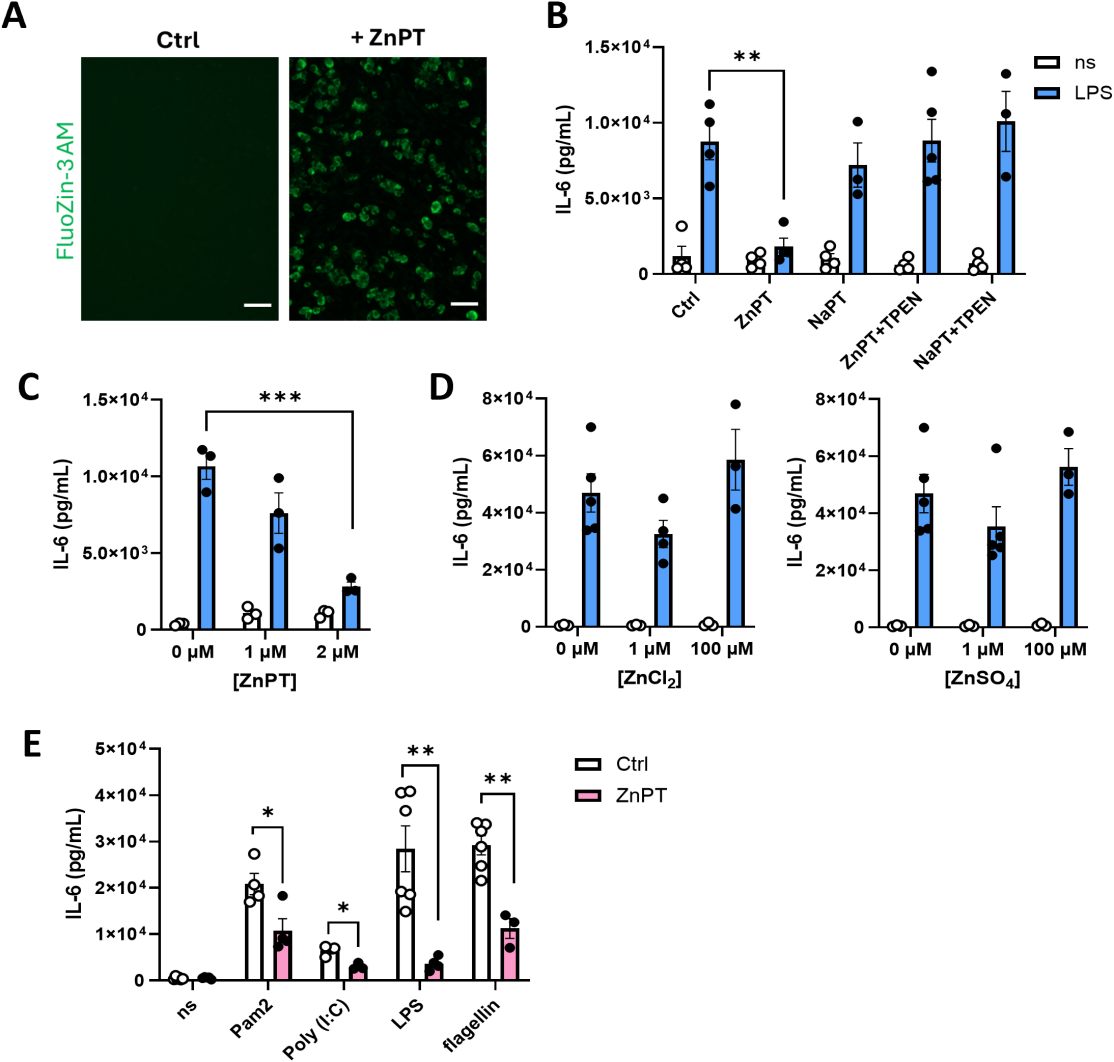
Zinc pyrithione restricts IL-6 production in adipocytes. **(A)** Increased intracellular Zn levels in zinc pyrithione (ZnPT)-treated primary adipocytes shown by Fluozin-3 AM staining. White scale bars indicate 100 µm. **(B–E)** IL-6 levels in the primary mouse adipocyte culture media measured by cytokine ELISA. **(B)** Reduction in IL-6 levels upon LPS stimulation when treated with ZnPT. The specificity of Zn effect was tested by evaluating the effects of sodium pyrithione (NaPT) and Zn chelator (TPEN) on IL-6 levels. **(C)** The effect of varying ZnPT concentrations on adipocyte IL-6 production upon LPS stimulation. **(D)** Effect of different levels of zinc chloride (left panel) and zinc sulfate (right panel) on adipocytes’ capacity to produce IL-6. **(E)** The effect of ZnPT treatment on adipocytes stimulated with different TLR ligands. ns, non-stimulated. Bars and error bars represent means ± SEM, circles represent independent repeats. Asterisk indicates statistical significance by Student’s *t*-test (*p<0.05, **p<0.01, ***p<0.001).

Given the ability of adipocytes to respond to TLR stimulation and the established role of Zn in immune regulation (27), we next examined whether Zn could impact adipocyte cytokine production. Primary mouse adipocytes were treated with Zn in the form of zinc pyrithione (ZnPT), and intracellular free Zn level was visualized using Zn-selective probe FluoZin-3 AM. FluoZin-3 AM loaded cells exhibited robust increase in fluorescent signals upon ZnPT exposure, confirming rapid elevation of intracellular zinc levels (**Figure 2A**). Adipocytes treated with ZnPT showed marked reduction in the level of IL-6 release upon LPS stimulation compared to controls (**Figure 2B**). This effect was specific to Zn, as treatment with sodium pyrithione (NaPT) did not modify LPS-driven IL-6 production in adipocytes. Addition of TPEN, a known Zn chelator, effectively reversed the suppressive effect of ZnPT on LPS-driven IL-6 production by adipocytes. Treatment with TPEN alone did not alter LPS-induced IL-6 production (data not shown). Further, ZnPT had a dose-dependent effect in suppressing the LPS-driven IL-6 production by adipocytes (**Figure 2C**). As pyrithione facilitates the direct movement of Zn into the cell cytosol circumventing the regulatory control of Zn flux by the Zn transporters, we next questioned whether Zn exposure, in the absence of an ionophore, would have a comparable effect. The administration of Zn in the form of zinc chloride (ZnCl_2_) or zinc sulfate (ZnSO_4_), failed to alter adipocytes’ ability to produce IL-6 in response to LPS stimulation (**Figure 2D**), implicating the importance of Zn cytosolic functions in regulating adipocyte IL-6 release.

Given the adipocytes’ ability to produce IL-6 in response to various TLR stimuli, we next investigated whether Zn similarly limits adipocyte IL-6 production in response to TLR stimulation beyond the LPS/TLR4 axis. Different TLR ligands were employed to stimulate the adipocytes treated with or without ZnPT. Adipocytes treated with ZnPT, followed by stimulation with Pam2, Poly(I:C), or flagellin produced significantly reduced amount of IL-6 (**Figure 2E**). Collectively, these data suggest that elevated intracellular level of bioavailable Zn in primary mouse adipocytes restricts cellular capacity to produce IL-6 following TLR stimulation.

**Figure 3:**
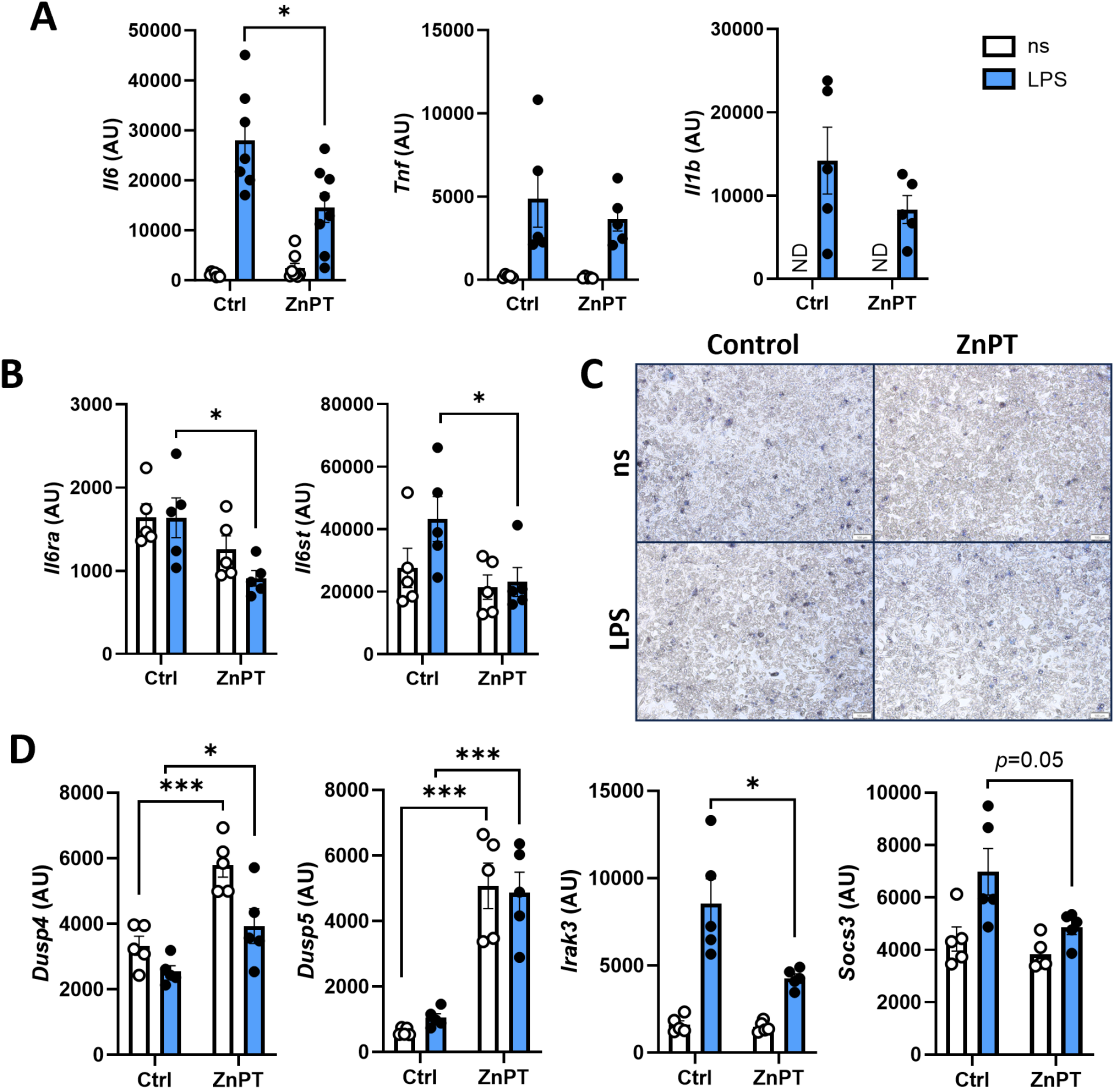
ZnPT alters the expression of genes involved in IL-6 production without inducing adipocyte cell death. mRNA levels of proinflammatory cytokines **(A)**, IL-6 receptors **(B)**, and suppressors of signaling pathways involved in IL-6 production **(D)** from primary mouse adipocytes treated with or without ZnPT, stimulated with LPS. Quantified by RT-qPCR, normalized to *Actb*. **(C)** Representative images of Trypan blue staining of cells after each treatment condition. White scale bars indicate 100 µm. ns, non-stimulated; ND, not detected. Bars and error bars represent means ± SEM, circles represent independent repeats. Asterisk indicates statistical significance by Student’s *t*-test (*p<0.05, ***p<0.001).

We next examined the potential mechanisms underlying Zn suppressive effects on IL-6 production in adipocytes. Cytokine production is often regulated at the level of transcriptional regulation (45). Therefore, we first examined the impact of Zn treatment on IL-6 mRNA expression. Adipocytes treated with ZnPT prior to LPS stimulation showed significantly lower *Il6* expression (**Figure 3A**). This was somewhat specific to IL-6, as genes of other inflammatory cytokines which are also strongly induced in adipocytes upon LPS stimulation (e.g., *Tnf*, *Il1b*) were not impacted by Zn treatment. We also asked if the mRNA levels of IL-6 receptor complex, IL-6R and gp130, were impacted by Zn treatment. The expression of *Il6ra* and *Il6st*, which encode IL-6R and gp130, respectively, were both reduced in LPS-stimulated adipocytes when treated with ZnPT (**Figure 3B**). These data suggest that Zn preferentially downregulates the mRNA transcription of IL-6, IL-6R, and gp130.

### 3.3 Zinc interferes with the activation of Erk1/2 and Stat3 signaling pathways

Although Zn is recognized for its various antioxidant roles (46), excess Zn can act as a prooxidant that promotes oxidative stress, leading to disruption of cell homeostasis (46). As ZnPT treatment may cause Zn overload we examined whether it impacts adipocyte survival. To rule out cytotoxicity of Zn overload as the cause of reduced IL-6 production, cell death was visually assessed with Trypan Blue viability staining and by the expression of genes important in cell death and survival. No visible difference in cell morphology nor in the number of dead cells was observed in adipocytes treated with ZnPT (**Figure 3C**). Further, genes indicative of apoptosis (*Casp3*, *Casp9*, and *Bim*) were not differentially expressed in LPS-stimulated adipocytes treated with or without ZnPT. In fact, ZnPT treatment of adipocytes resulted in elevated expression of anti-apoptotic gene (*Bcl2*) (**Supplementary Figure 2**). These data suggest that induction of cell death is an unlikely process associated with ZnPT-dependent restriction of LPS-driven IL-6 production in adipocytes.

We next investigated if ZnPT-dependent reduction of adipocyte IL-6 production is linked to transcriptional downregulation of TLRs, resulting in reduced stimulation signals in adipocytes. To determine if elevated intracellular Zn modulates TLR expression, the levels of *Tlr* mRNAs were compared between ZnPT-treated and untreated adipocytes. Surprisingly, adipocytes stimulated with LPS exhibited increased *Tlr4* expression when exposed to ZnPT compared to untreated controls (**Supplementary Figure 3**). *Tlr2*, *Tlr3*, and *Tlr5* expression levels were not significantly altered by ZnPT treatment in both non-stimulated and stimulated conditions with their corresponding ligands (Pam2, poly(I:C), and flagellin, respectively). These data suggest that differences in adipocyte *Tlr* expression is likely not the dominant process responsible for Zn-induced reduction in IL-6 secretion in adipocytes.

Extracellular signals received by TLRs are transduced within the cells through multiple signaling cascades, including the activation of NF-κB, MAPKs, and Jak/Stat pathways, which in turn induce the expression of inflammatory cytokines such as IL-6 (47). Various regulatory molecules impact the activation or suppression of these signaling pathways, resulting in differential expression of downstream genes. Thus, to examine which inflammatory signaling pathways may be influenced by ZnPT treatment leading to altered expression of IL-6 and its receptor genes, the expression of several regulators known to impact the inflammatory signaling pathways were quantified. Dual-specificity phosphatases (Dusps) are negative regulators of MAPK pathway through MAPK dephosphorylation, with Dusp5 specifically targeting Erk1/2 (48). IL-1 receptor-associated kinase 3 (Irak3) inhibits Myd88-mediated signaling (49), while suppressor of cytokine signaling 3 (Socs3) is a member of Stat-induced Stat inhibitor family and serves as both a regulator and a target of Stat signaling pathways (50). Significant increases in *Dusp4* and *Dusp5* expression was observed in adipocytes exposed to ZnPT, whereas *Irak3* and *Socs3* expression was found to be decreased (**Figure 3D**). *Dusp5* was robustly induced with ZnPT treatment (~5-fold increase on average) both in unstimulated and LPS-stimulated cells.

**Figure 4:**
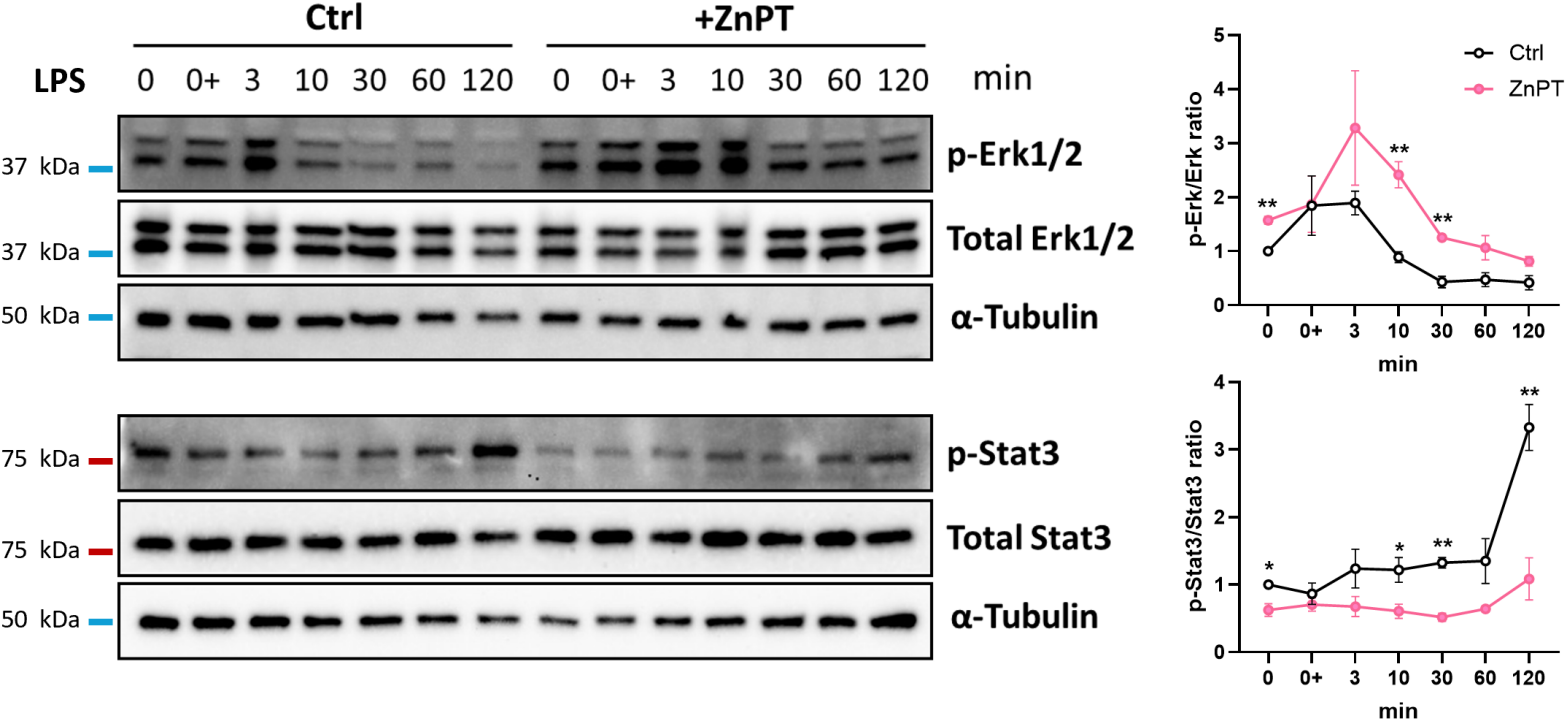
ZnPT interferes with the activation of Erk1/2 and Stat3 signaling pathways. Proteins of intracellular signaling pathways quantified by Western blot analysis. Phosphorylation levels of Erk1/2 and Stat3 proteins were assessed. Primary mouse adipocytes were collected at indicated timepoints after LPS stimulation, in the presence or absence of ZnPT. Left panels, representative image of 3 biological repeats. α-Tubulin included as loading control. The positions of molecular size markers are shown on the left of each blot. Right panels, ratio of phospho-protein over total protein indicative of signaling activation, with values presented relative to Ctrl at 0 min timepoint. Circles and error bars represent means ± SEM. Asterisk indicates statistical significance (*p<0.05, **p<0.01) between Ctrl and ZnPT at each time point, determined by multiple unpaired *t*-tests with FDR of *q* < 0.05.

Given the elevated *Dusp4*/*5* expression in adipocytes treated with ZnPT, we next examined if increased intracellular Zn levels suppress Erk1/2 activation by upregulating *Dusp* expression. Activation of Erk1/2 following LPS stimulation was assessed in adipocytes treated with or without ZnPT. Contrary to our anticipation, p-Erk1/2 to total Erk1/2 ratio was higher in adipocytes treated with ZnPT, revealing stronger Erk1/2 activation (**Figure 4**, upper panels). Instead, we found attenuated activation of Stat3, as indicated by lower p-Stat3/Stat3 ratio in ZnPT treated adipocytes (**Figure 4**, lower panels). Together, these results suggest that intracellular Zn availability differentially impacts inflammatory signaling pathways downstream of TLR4 in primary mouse adipocytes.

### 3.4 Zinc restricts IL-6-Stat3 positive feedback loop

**Figure 5:**
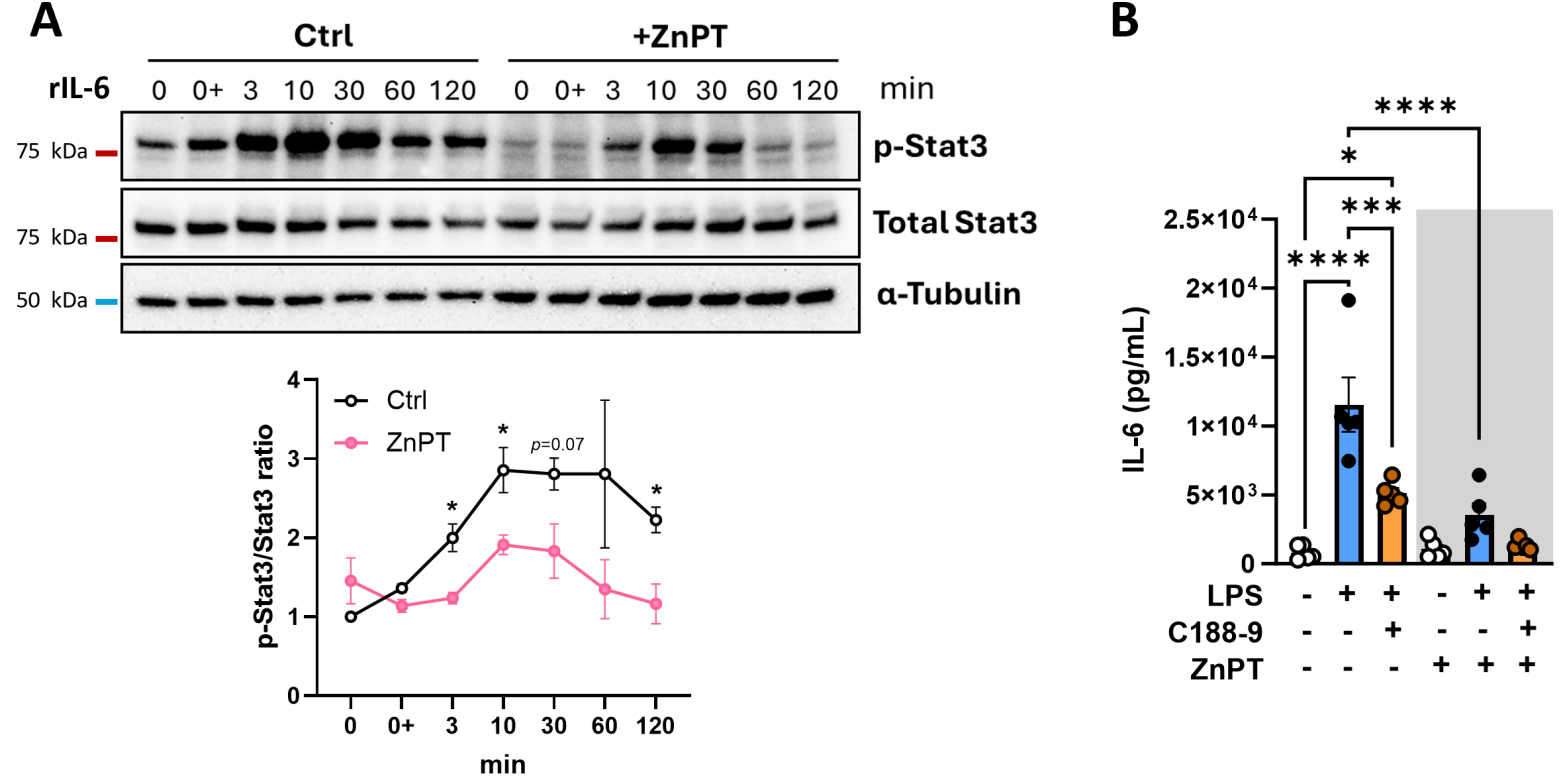
ZnPT restricts IL-6 induced activation of Stat3 signaling in adipocytes. **(A)** Protein levels of phospho-Stat3 and total Stat3 quantified by Western blot analysis. Primary mouse adipocytes were collected at indicated timepoints after treatment with recombinant IL-6, in the presence or absence of ZnPT. Upper panel, representative image of 3 biological repeats. α-Tubulin included as loading control. The positions of molecular size markers are shown on the left of each blot. Lower panel, ratio of phospho-Stat3 over total Stat3 indicative of signaling activation, with values presented relative to Ctrl at 0 min timepoint. Circles and error bars represent means ± SEM (*p<0.05, Ctrl vs ZnPT at each time point). **(B)** IL-6 levels in the culture media of primary adipocytes upon LPS stimulation when treated with Stat3 inhibitor (C188-9), in the presence or absence of ZnPT, measured by cytokine ELISA. Bars and error bars represent means ± SEM, circles represent independent repeats. Asterisk indicates statistical significance by one-way ANOVA followed by Bonferroni *post hoc* analysis (*p < 0.05, ***p<0.001, ****p<0.0001).

Activation of Stat3 develops in response to a range of extracellular stimuli transmitted via TLRs, cytokine receptors, receptor tyrosine kinases, G protein-coupled receptors, and growth factor receptors (51). Regardless of ZnPT treatment, activation of Erk1/2 occurred within 3 minutes post-LPS stimulation, preceding Stat3 activation by approximately 2 hours (**Figure 4**). We queried if the delay in Stat3 activation occurred through the skewing of the positive feedback loop rather than by direct TLR-mediated route, in which IL-6 production drives the activation of IL-6 receptor-mediated pathway. To examine this, recombinant IL-6 (rIL-6) was used to confirm that Stat3 is activated in adipocytes in response to IL-6, and to define the timeframe of Stat3 activation following IL-6 exposure. Indeed, rIL-6 stimulation was sufficient to activate Stat3 at a much earlier point (10 min) compared to LPS stimulation (**Figure 5A**, Ctrl). Subsequently, we analyzed the contribution of Stat3 activation in adipocytes stimulated with LPS. Adipocytes were mock-treated or treated with Stat3 inhibitor (C188-9) and stimulated with LPS. Inhibition of Stat3 resulted in a significant, although not complete, reduction of LPS-driven IL-6 production by adipocytes (**Figure 5B**, white background). These data demonstrate that while primary mouse adipocytes with inhibited Stat3 can still produce IL-6, Stat3 activation is required to achieve maximal IL-6 production via a positive feedback loop in response to TLR stimulation.

We questioned how Zn may influence the TLR/Stat3/IL-6 signaling axis and the IL-6/Stat3 positive feedback loop. First, we examined the rIL-6-induced Stat3 activation in ZnPT-treated adipocytes. The level of IL-6 produced by ZnPT-treated cells was evaluated after LPS stimulation with or without the Stat3 inhibitor. Consistent with our previous observation, the Stat3 activation was markedly reduced in adipocytes exposed to ZnPT prior to rIL-6 stimulation (**Figure 5A**), implying a role for increased intracellular Zn in attenuating the Stat3-driven amplification of IL-6 production. Notably, adipocyte treatment with ZnPT and Stat3 inhibitor did not further reduce IL-6 production from ZnPT alone. These data suggest that ZnPT effects on LPS-driven IL-6 production are possibly mediated by suppression of Stat3 signaling (**Figure 5B**, grey background). Combined, these data demonstrate that adipocyte production of IL-6 is maximized by a Stat3-driven amplification loop, and that elevated intracellular Zn restricts IL-6 production by suppressing the IL-6/Stat3 positive feedback loop.

## 4 Discussion

**Figure 6:**
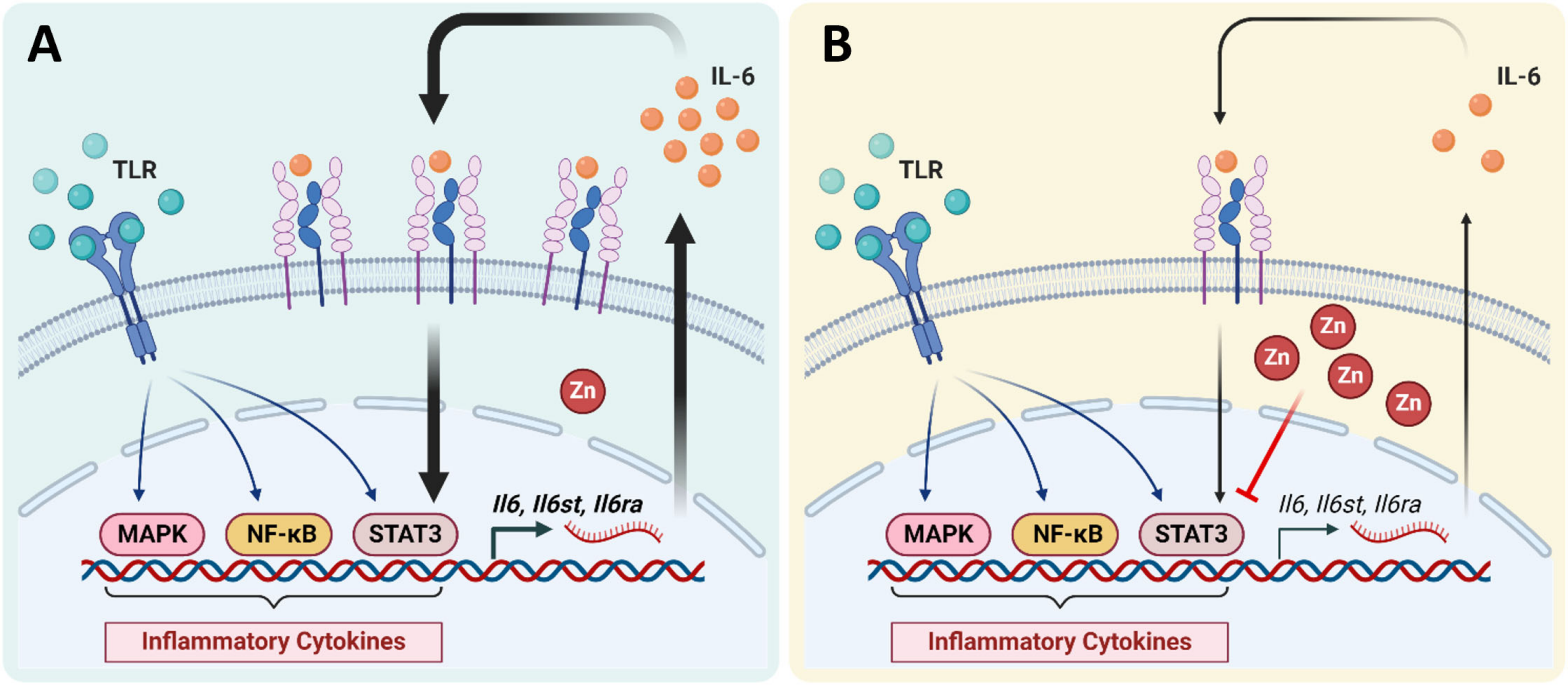
Schematic diagram illustrating the proposed mechanism of adipocyte IL-6 production **(A)** and its suppression by the presence of high zinc **(B)**.

In this study, we demonstrated that the treatment of ZnPT to primary mouse adipocytes effectively suppresses their IL-6 production. Specifically, we confirmed that adipocytes, like immune cells, respond to various immunological stimuli through activation of TLR signaling and produce IL-6. We showed that IL-6 production by adipocytes is maximized via a Stat3-mediated positive feedback loop. The initially released IL-6 acts in an autocrine manner to activate IL-6 receptors, which subsequently triggers Stat3 activation further enhancing IL-6 release. Increased cellular influx of Zn, mediated by pyrithione, suppressed IL-6-induced Stat3 activation as well as the delayed Stat3 activation following LPS stimulation. These data suggest that increased Zn influx into adipocytes dampens the amplification loop thereby reducing total IL-6 production. A schematic diagram illustrating the proposed mechanism of IL-6 production in adipocytes, and the suppressive role of Zn in this setting, is shown in **Figure 6**.

The initial rise in IL-6 levels is necessary to activate Stat3, which likely occurs through TLR-activated signaling pathways independent of Stat3. For example, early activation of Erk1/2 was observed following LPS stimulation. Adipocytes were still able to produce substantial amounts of IL-6 even when Stat3 activation was inhibited, implying that alternative inflammatory pathways, such as the MAPK pathway, drive the initial IL-6 production. In fact, we observed immediate activation of Erk1/2 upon LPS stimulation, both in the presence and absence of ZnPT, supporting this notion. Alternatively, it is possible that a molecule other than IL-6 serves as the inducer of Stat3 signaling. Stat3 can be activated by numerous factors, including cytokines from the common gamma chain, gp130, and IL-10 receptor families, as well as type I and II interferons and various growth factors (51, 52). Adipocytes are known to secrete more than 50 cytokines and adipokines, some of which (e.g. IL-6, TNFα, IL-1β) could be released upon LPS stimulation and potentially trigger Stat3 signaling (53). Our data show that LPS induces the expression of *Tnf* and *Il1b* in adipocytes, although the corresponding protein levels in media were not measured. While we verified Stat3 activation in response to IL-6, we did not rule out the possibility of activation by other adipocyte-derived factors, such as TNFα and IL-1β.

The observed increase in *Tlr4* expression in ZnPT-treated adipocytes, despite suppressed IL-6 release, may be attributed to multiple factors. One possibility is the elevated Erk signaling in Zn-loaded adipocytes. Erk is a known inducer of the c-Fos/c-Jun pathway, and as the *Tlr4* promoter contains a functional AP-1 binding site (54), this signaling axis could directly enhance *Tlr4* transcription. However, further validation of this mechanism was not performed, nor was it verified whether this mRNA increase translated to higher protein levels, warranting future investigations on this topic.

Zn is an important regulator of cellular signaling (25), and its effects on the modulation of protein kinases have been explored across various cell types. Evidence suggests that Zn influences MAPK signaling, with an increase in Erk1/2 phosphorylation reported in fibroblasts (55) and neurons (56, 57) treated with Zn. Activation of Erk and Akt pathways by elevated intracellular Zn has also been reported in in HeLa, MCF10A, and HT-22 cells (58). Our data further demonstrate that zinc treatment enhances Erk signaling in adipocytes as well. However, the specific protein(s) affected by Zn and the exact mechanism of action remain unknown. It is proposed that Zn targets signaling upstream of Ras, thereby impacting Erk (58), and may modulate phosphatases that regulate the kinase cascade (59, 60). While Zn treatment increased Erk1/2 phosphorylation, it suppressed Stat3 phosphorylation and consequently attenuated the Stat3/IL-6 amplification loop in adipocytes. Published data suggests differential effects of Zn on Stat signaling pathway. Specifically, in rats with maternal Zn deficiency, Stat1 and Stat3 signaling were impaired in the fetal brain (61). The mammary epithelial cells of ZnT2^-/-^ mice were found to accumulate cytosolic Zn as a consequence of impaired Zn efflux, which resulted in diminished Stat5 phosphorylation in these cells (62). Mechanistically, Zn activated SHP1, a protein tyrosine phosphatase that acts as a negative regulator of Stat3 pathway, to inhibit Stat3 activation in hepatocytes (63). Although this mechanism has not been tested in adipocytes, it is conceivable that Zn-driven suppression of Stat3 signaling in adipocytes may involve a similar pathway.

Zn homeostasis is crucial for maintaining cellular health and metabolic function. While Zn is essential, excessive levels of Zn can lead to toxicity (46, 64). Therefore, the cellular flux of Zn is tightly regulated by the expression of multiple Zn transporters of ZnT and ZIP families distributed across different cellular compartments (65). Additionally, cells express metallothioneins that bind free Zn, minimizing the level of reactive Zn and releasing it as needed to mitigate the toxicity (66). Zn availability is sensed by the cells through the metal regulatory transcription factor (MTF-1) (67). MTF-1 has a DNA-binding domain with six Zn fingers, two of which have low affinity to Zn, allowing it to respond effectively to fluctuating Zn levels. Under conditions of high Zn, active MTF-1 interacts with metal-responsive elements (MREs) located in the promoters of target genes, orchestrating their expression to maintain homeostasis. For example, the expression of ZnT1 Zn efflux transporter is induced to facilitate the removal of excess intracellular Zn. It is important to note that only a limited number of genes are direct targets of MTF-1 (67); many genes that respond to Zn levels are regulated indirectly or through mechanisms that remain to be elucidated. In our study, when Zn was introduced in the form of ZnCl_2_ or ZnSO_4_, no significant changes in IL-6 levels produced by LPS-stimulated adipocytes were observed. We speculate that such outcomes reflect the above-described cells’ ability to regulate Zn flux. As a charged transition metal ion, Zn cannot pass through biological membranes freely, and instead cells rely on membrane Zn transporters for its import and export. Upon exposure to high Zn levels, rapid internalization and degradation of ZIP transporters is triggered which effectively blocks Zn influx, while increased expression of ZnT1 promotes Zn efflux. Together these processes work in unison to minimize the impact of sudden changes in Zn levels (65).

Pyrithione is a membrane permeable ionophore that, when combined with Zn to form Zn pyrithione, allows movement of Zn across biological membranes. This mechanism circumvents the cells’ regulation and can directly promote greater influx of Zn, potentially elevating intracellular Zn concentrations. In our study, we utilized ZnPT at a concentration of 2 µM. Considering that total cellular Zn concentrations are estimated to be in the tens to hundreds micromolar range (65), it may not seem high. However, since cells actively strive to maintain labile Zn low [estimated in picomolar to nanomolar range (68, 69)], the concentration of ZnPT used could be substantial. Intracellular Zn overload can induce cell death by activating Bim, which triggers Caspase-3-dependent apoptosis in cultured neurons (70). Therefore, we postulated that the Zn concentration might be too high, potentially disrupting Zn homeostasis and leading to cell death and the inability to produce cytokines. To evaluate this, we measured the expression of apoptosis-related genes including *Bim* and *Casp3*. Our data found no notable differences between LPS-stimulated cells with and without ZnPT exposure. Given the reported nearly 3-fold increase in *Bim* mRNA expression during Zn-induced apoptosis (70), it is unlikely that ZnPT treatment in our experiments was sufficient to trigger apoptosis. Alternatively, oxidative stress induced by excess Zn can also drive necroptosis or activate mitochondrial and lysosomal autophagy pathways, leading to different forms of cell death (64). However, our analysis of cell viability using Trypan blue staining found no visible differences between untreated and ZnPT treated adipocytes, confirming that the observed decrease in cytokine production was likely not attributable to zinc toxicity.

In our experiments, ZnPT treatment led to an increase in *Dusp5* expression in adipocytes, which encodes a known negative regulator of the Erk signaling pathway. Consequently, we initially anticipated a reduction in Erk activation in the ZnPT-treated cells. Contrary to our expectations, however, LPS stimulation led to higher Erk1/2 phosphorylation in ZnPT-treated adipocytes than in untreated counterparts. We speculate that this observation may be attributable to the negative feedback mechanism within the Erk signaling pathway. While Dusp5 serves as a regulator of Erk signaling, it is also a target and a substrate of Erk. Specifically, Erk activation induces *Dusp5* mRNA expression, and Erk-induced Dusp5 phosphorylation stabilizes the protein and increases its half-life (71). These processes together enhance the regulatory effects of Dusp5, leading to the resolution of Erk activation. Since the mRNA levels were measured in cells collected 4 hours after LPS stimulation, the elevated *Dusp5* expression may be a result of prior Erk activation that occurred earlier in the timeline. In line with this, we also observed a reduction in LPS-induced *Socs3* expression in ZnPT-treated adipocytes. As with Dusp5 and Erk signaling, Socs3 is a suppressor of Stat signaling pathway whose expression is induced by active Stat3, forming a negative feedback loop that controls cytokine production (50). The reduced *Socs3* expression is therefore presumed to result from Stat3 downregulation, rather than an indication of increased (i.e. less suppressed) Stat3 activity.

Indeed, persistently high levels of *Dusp4* and *Dusp5* expression has been recognized as adverse indicators in obesity and insulin resistance, as they are linked to increased inflammation (72). In this study, although ZnPT treatment led to increased *Dusp5* expression and enhanced LPS-induced Erk activation, adipocyte IL-6 release was significantly reduced along with diminished activation of Stat3. These findings suggest that increase in intracellular Zn can suppress IL-6 production by modulating Stat3 pathway, apart from elevated Erk signaling. Further, our data suggest that intracellular Zn availability could be a key factor in determining the inflammatory impact of adipocytes in obesity. One of the effects of IL-6 is to restrict Zn availability by inducing the expression of metallothionein (73), which binds and sequesters labile Zn. This is advantageous during an acute immune response, as it deprives the pathogens of the essential trace metal thereby limiting their proliferation (74). In a chronic inflammatory state (e.g., obesity) with consistently high IL-6 levels, however, such effects of IL-6 may feed into the sustained and unresolved IL-6 production by adipocytes. Our findings suggest that replenishing Zn in adipocytes suppresses IL-6 release, which can potentially facilitate the restoration of cellular Zn homeostasis and normalizing the amplified IL-6 production.

The identification of Zn as a negative regulator of adipocyte IL-6 production offers a mechanistic rationale for the clinical benefits observed in Zn supplementation trials. As noted, adipocyte-derived IL-6 promotes ATM accumulation and adipose tissue inflammation (17). This is particularly critical in obesity setting, given that adipose tissue inflammation is a known driver of metabolic complications, and that IL-6 specifically has been implicated in hepatocyte insulin resistance (6), atherosclerotic plaque growth (7), and the differentiation (8) and sustenance (9) of the pathogenic Th17 cells, the primary culprits in MASH development (10). While clinical studies have demonstrated that replenishing Zn can lower serum IL-6 and improve outcomes in metabolic syndrome (31–33) and MASH (36), the precise molecular mechanisms of this intervention have remained elusive. Our findings suggest that these systemic benefits may stem, in part, from the ability of zinc to disrupt the Stat3-mediated positive feedback loop, thereby limiting adipocyte IL-6 production.

It is important to note that different adipose tissue depots exhibit distinct physiological profiles (75). While this study utilized iWAT-derived adipocytes to ensure experimental consistency, future studies utilizing alternative *ex vivo* models to explore depot-specific differences in Zn sensitivity would be valuable. Additionally, while this study centered on the signaling pathways governing primary mouse adipocyte IL-6 production, expanding the analysis to determine the impact of Zn on the broader adipokine profile including anti-inflammatory cytokines and to reproduce these findings in primary human adipocytes are warranted. Furthermore, *in vivo* studies are required to determine whether intracellular Zn levels are intrinsically altered within obese adipocytes, and if dietary Zn regimens can achieve the intracellular thresholds required to interrupt the Stat3-mediated amplification loop in lean and obese states.

In summary, we investigated the impact of Zn on adipocyte inflammatory vigor. We show that ZnPT treatment restricts TLR signaling-driven IL-6 production in primary mouse adipocytes, via suppressing Stat3 signaling activation and thereby constraining the positive feedback loop of amplifying IL-6 production. Our findings demonstrate a novel inhibitory role of Zn in the IL-6 production of non-immune cells known to contribute to disease pathogenesis. Specifically, our findings suggest a potential role of Zn in the regulation of adipocyte-associated chronic inflammation (76). Thus, adipocyte dysfunction driven by Zn imbalance may be an unexplored yet critical factor in chronic inflammation.

## Data Availability

The RNA-seq dataset used in this study has been deposited in GEO database and can be accessed at GSE110236 [https://www.ncbi.nlm.nih.gov/geo/query/acc.cgi?].
