# Supplementary material for: Zinc suppresses Stat3-driven IL-6 production in primary mouse adipocytes
Source: Front Immunol. 2026 Feb 9;17:1714168. doi: 10.3389/fimmu.2026.1714168 (PMC12926143; doi:10.3389/fimmu.2026.1714168)
Supplement: Supplementary file 1 [file DataSheet1.docx]

Supplementary Material


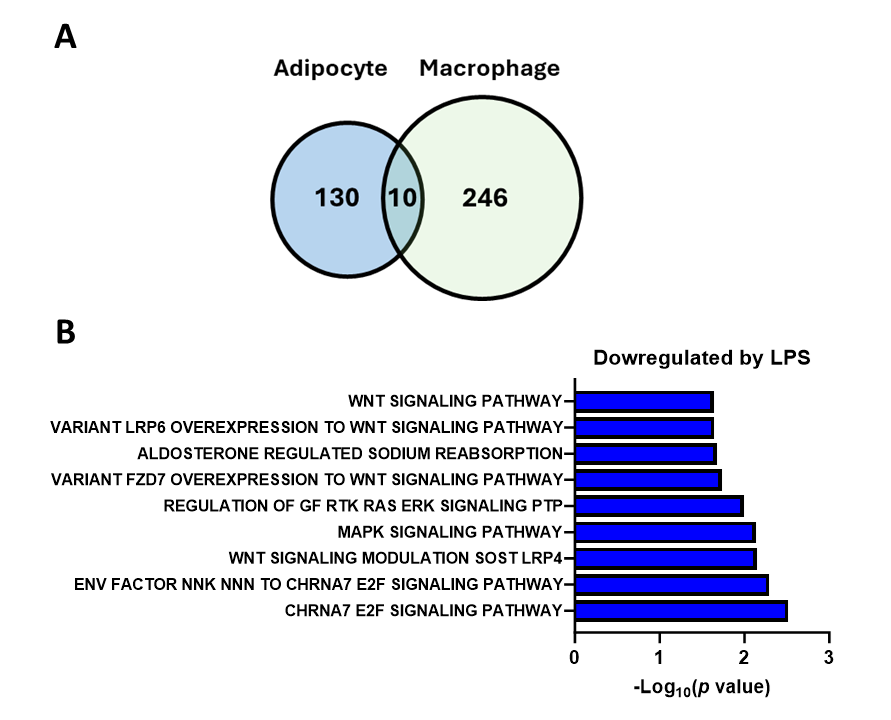


**Supplementary Figure 1. TLR-driven downregulation of genes in adipocytes.**

**(A)** From the gene expression analysis reported in Figure 1A, the number of downregulated genes in either adipocytes, macrophages, or both are indicated. **(B)** ToppGene pathway enrichment analysis of the 10 co-downregulated genes in LPS-stimulated adipocytes and macrophages using KEGG pathways for mouse. Full list of differentially expressed genes is provided in Data Sheet 2.

**Supplementary Figure 2. ZnPT treatment of primary mouse adipocytes does not alter expression of genes associated with cell death.**

mRNA levels of respective genes indicative of cell death (*Casp3*, *Casp9*, *Bim*) and survival (*Bcl2*) in primary mouse adipocytes treated with or without ZnPT, stimulated with LPS. Quantified by RT-qPCR, normalized to *Actb*. ns, non-stimulated. Bars and error bars represent means ± SEM, circles represent independent repeats. Asterisk indicates statistical significance by Student’s *t*-test (**p<*0.05).


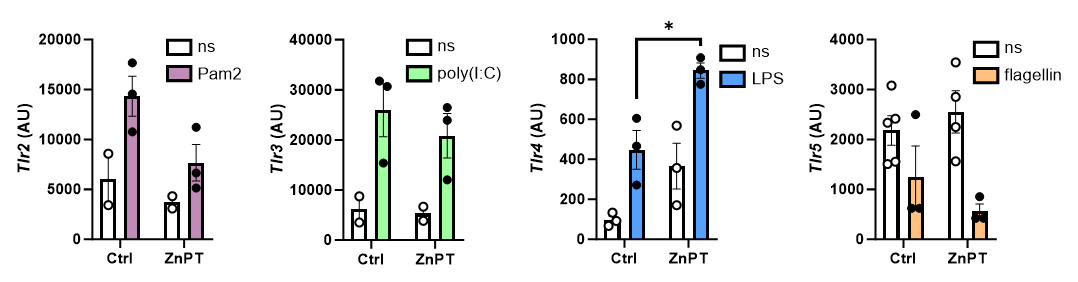


**Supplementary Figure 3. Suppression of IL-6 by ZnPT does not correlate with downregulation of *Tlr* gene expression in primary mouse adipocytes.**

mRNA levels of *Tlr2, Tlr3, Tlr4* and *Tlr5* in primary mouse adipocytes treated with or without ZnPT, stimulated with respective TLR ligands. Quantified by RT-qPCR, normalized to *Actb*. ns, non-stimulated. Bars and error bars represent means ± SEM, circles represent independent repeats. Asterisk indicates statistical significance by Student’s *t*-test (**p<*0.05).

**Supplementary Table 1. List of primer pairs used in the study**

| Gene | Forward (5’-3’) | Reverse (5’-3’) |
| --- | --- | --- |
| *Actb* | GGCCCAGAGCAAGAGAGGTA | GGTTGGCCTTAGGTTTCAGG |
| *Casp3* | CTGACTGGAAAGCCGAAACTC | CGACCCGTCCTTTGAATTTCT |
| *Casp9* | GGTCGTTAAACCCCTAGACCA | TGACGGGTCCAGCTTCACTA |
| *Bcl2* | AGTACCTGAACCGGCATCTG | AGGGTCTTCAGAGACAGCCA |
| *Bim* | CCCGGAGATACGGATTGCAC | GCCTCGCGGTAATCATTTGC |
| *Il6* | TGGTACTCCAGAAGACCAGAGG | AACGATGATGCACTTGCAGA |
| *Tnf* | CCAGACCCTCACACTCAGATCA | CACTTGGTGGTTTGCTACGAC |
| *Il1b* | TGTGCTCTGCTTGTGAGGTGCTG | CCCTGCAGCTGGAGAGTGTGGA |
| *Il6st* | TTACTACGTGAATGCCAGCTACA | GACGTGGTTCTGTTGATGACA |
| *Il6ra* | GCCACCGTTACCCTGATTTG | TCCTGTGGTAGTCCATTCTCTG |
| *Tlr2* | CACCAAGATCCAGAAGAGCC | TAGGGCTTCACTTCTCTGC |
| *Tlr3* | CTCCTCTTGAACAACGCCCA | GGA ACCGTTGCCGACATC |
| *Tlr4* | CATCCAGGAAGGCTTCCACA | GGCGATACAATTCCACCTGC |
| *Tlr5* | TGCTCTCTCCGGGCAGTTTA | GCCGAACAGGGTGACGTT |
| *Dusp4* | CATCGAGTACATCGACGCAG | ATGAAGCTGAAGTTGGGCGA |
| *Dusp5* | GCACCACCCACCTACACTAC | CCTTCTTCCCTGACACAGTCAAT |
| *Irak3* | GAGAATTGCTCTGGTCCTGGG | CACCTCAAGTGGGAAGCTGG |
| *Socs3* | CACAGCAAGTTTCCCGCCGCC | GTGCACCAGCTTGAGTACACA |
